# Room temperature processed high mobility W-doped In_2_O_3_ electrodes coated via in-line arc plasma ion plating for flexible OLEDs and quantum dots LEDs

**DOI:** 10.1038/s41598-018-30548-w

**Published:** 2018-08-13

**Authors:** Jae-Gyeong Kim, Ji-Eun Lee, Sung Min Jo, Byung Doo Chin, Ju-Yeoul Baek, Kyung-Jun Ahn, Seong Jun Kang, Han-Ki Kim

**Affiliations:** 1 School of Advanced Materials Science and Engineering, Sungkyunkwan University, 2066 Seobu-ro, Jangan-gu, Suwon, Gyeonggi-do 16419 Republic of Korea; 2 Department of Polymer Science and Engineering, Dankook University, 152 Jukjeon-ro, Suji-gu, Yongin-si, Gyeonggi-do 16890 Republic of Korea; 3 SNTEK Co., Ltd, 1433-100, Seobu-Ro, Gwonseongu, Suwon-Si, Gyeonggi-do 16648 Republic of Korea; 4 Department of Advanced Materials Engineering for Information and Electronics, Kyung-Hee University, 1 Seochen-dong, Yongin-si, Gyeonggi-do 446-701 Republic of Korea

## Abstract

We fabricated W-doped In_2_O_3_ (IWO) films at room temperature on a flexible PET substrate using an in-line arc plasma ion plating system for application as flexible transparent conducting electrodes (FTCEs) in flexible organic light emitting diodes (OLEDs) and quantum dots light emitting diodes (QDLEDs). Due to the high-energy flux of the sublimated ions generated from the plasma region, the IWO films showed a well-developed crystalline structure with a low sheet resistance of 36.39 Ohm/square and an optical transmittance of 94.6% even though they were prepared at room temperature. The low sheet resistance of the IWO film processed at room temperature is attributed to the high mobility (59 cm^2^/V-s) in the well-developed crystalline structure of the ion-plated IWO film and screening effect of W dopants. In addition, the better adhesion of the ion-plated IWO film on the PET substrate led to small critical outer and inner bending radii of 6 and 3 mm, respectively, against substrate bending. Due to the low sheet resistance, high optical transmittance, better crystallinity, better adhesion, and outstanding flexibility of the ion-plated IWO films, the flexible OLEDs and QDLEDs with the IWO electrodes showed better performances than flexible OLEDs and QDLEDs with sputtered flexible ITO anodes. This indicates that in-line arc plasma ion plating is a promising large area coating technique to realize room temperature processed high-quality FTCEs for flexible OLEDs and QDLEDs.

## Introduction

Indium oxide (In_2_O_3_), zinc oxide (ZnO), and tin oxide (SnO_2_)-based transparent conducting oxide films have been commercialized as transparent conducting electrodes (TECs) in flat panel displays, photovoltaics, touch screen panels, transparent heaters, and transparent electronics due to their low resistivity, high optical transmittance, and ease of large area coating^1–9^. Among numerous degenerate transparent conducting oxide semiconductors, Sn-doped In_2_O_3_ (ITO) films coated on glass substrates at high process temperatures have been mainly employed as TCEs for optoelectronic devices in academic research and industry because sputtered ITO films possess a low resistivity (2–5 × 10^−4^ Ohm-cm) and high optical transmittance (80–85%)^10–12^. In addition, the ease of scalability and mature sputtering process techniques have realized ten-generation glass-sized ITO films. As a result, we believe that there is not a competitive sputtered ITO film for commercial organic light emitting diodes (OLEDs) and next generation quantum dot light emitting diodes (QDLEDs). However, ITO films prepared by a low-temperature sputtering process on a flexible substrate generally generate amorphous ITO films with a high resistivity due to the low mobility (10–15 cm^2^/V-s) of amorphous structured ITO films caused by severe scattering. Generally, severe scattering at ionized impurity, oxygen vacancies, and disordered structure led to low electron mobility in the amorphous ITO films. Although substrate heating or a post annealing process could be applicable to ITO films coated on a glass substrate, a high-temperature process is not applicable for general flexible substrates with a low glass transition temeprature^13–15^. To overcome the problems of low-temperature processed ITO films, carbon-based TCE, metal-nanostructured TCE, metal grid TCE, Ag network, and conducting polymer films have been extensively investigated^16–21^. However, each FTCE material still has critical problems to overcome to replace amorphous ITO electrodes such as the quality of TCE, scalability, possibility of mass production, and compatibility with the current device fabrication processes. Another promising FTCE scheme is high-mobility oxide TCEs such as InWO, InMoO, InZrO, InTiO, InNbO, InTaO, and InGeO films prepared by typical sputtering processes^22–28^. However, the high mobility oxide TCEs prepared by low-temperature sputtering also require a high-temperature annealing process to increase the carrier mobility because the kinetic energy of atoms sputtered on the flexible substrate is quite low. As a promising low-temperature coating process to prepare high-quality thin films on flexible substrates, an arc plasma ion plating technique has been suggested^13–15,29–33^. In the arc plasma ion plating process, the ionized atoms, which are generated in the high density plasma beam region, resulted in high-quality films without additional substrate heating due to the high kinetic energy of the accelerated ions to the self-biased substrate^29–33^. Therefore, even at low process temperatures, high-quality oxide TCE films could be deposited on flexible substrates using arc plasma ion plating. Huber *et al*. reported that plasma assistance during the ion plating process led to deposit high-quality ITO films with a resistivity of 4.4 × 10^−4^ Ohm-cm and an optical transmittance of 86% below a process temperature of 100 °C^33^. Niino *et al*. also reported the feasibility of DC arc discharge ion plating for the low-temperature deposition of ITO films on a SiO_2_-coated polycarbonate substrate^30^. Although the characteristics of ion-plated ITO and Ga-doped ZnO films have been well reported^30–33^, investigation of arc plasma ion-plated high mobility W-doped In_2_O_3_ (IWO) films for FTCEs and applications for flexible OLEDs and QDLEDs are still lacking. As we reported in the previous work, sputtered IWO films showed low resisviity comparable to ITO films due to high carrier mobility and high optical transmittance in visible wavelength region and near infrared wavelength region due to low carrier concentration^34^. In addition, with small W doping concentration of 1–2 wt%, the IWO fims showed a high optical transmittance and conductivity. In spite of various merits of IWO films, there is no report on electrical, optical, and mechanical properties of ion-plated IWO films for OLEDs and QDLEDs.

In this work, we comprehensively investigated the characteristics of IWO films prepared by a five-generation in-line arc plasma ion plating system at room temperature on flexible PET substrates. Using optimal IWO films, we compared the electrical, optical, structural, morphological, and mechanical properties of the ion-plated IWO films with sputtered ITO films to demonstrate their feasibility as a FTCE. In addition, we compared the performance of flexible OLEDs and flexible QDLEDs fabricated on ion-plated IWO and sputtered ITO films and suggested the potential of the arc plasma ion-plating technique to prepare high-quality FTCEs as well as the merit of ion-plated IWO films for next generation flexible OLEDs and QDLEDs.

## Results

**Figure 1:**
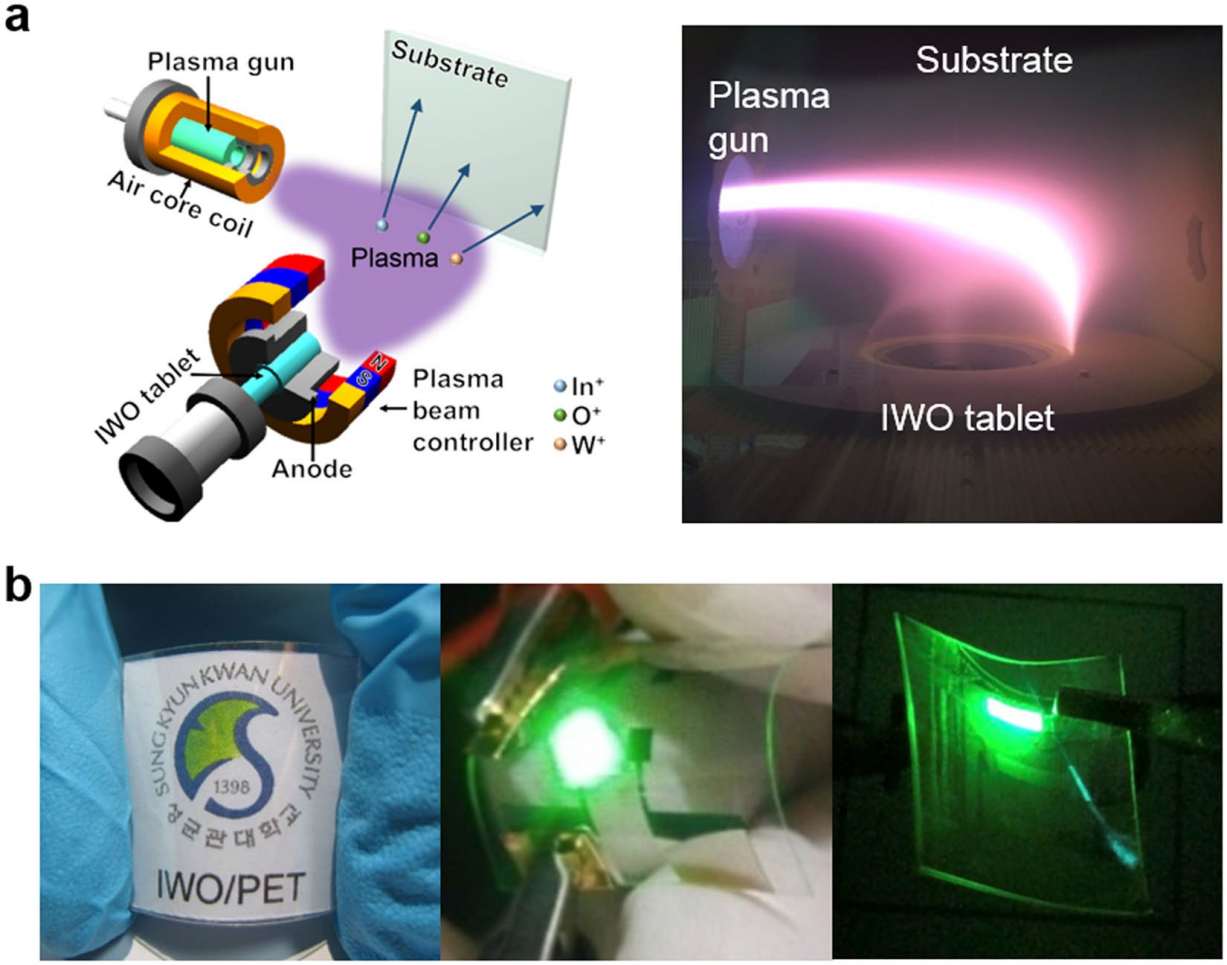
(**a**) Schematics of the in-line type arc plasma ion plating system employed for deposition of IWO films on PET substrates. A picture of the curved plasma beam irradiated on the IWO tablet facing the PET substrate. The picture was obtained from the top viewport in the chamber. (**b**) Pictures of the flexible IWO electrode on the PET substrate and flexible OLED and QDLED fabricated on optimized ion-plated IWO electrodes.

Figure 1a schematically illustrates the in-line arc plasma ion plating process used to deposit 100 nm thick IWO films on a 125 µm thick PET substrate at room temperature. Unlike a typical ion plating system where the plasma beam irradiated on the tablet is located at the bottom of a vacuum chamber, the plasma beam in the in-line arc plasma ion plating system (Sntek, Reactive plasma deposition system) is irradiated from the side to the vertically located IWO tablet which directly faces the PET substrate. The much higher electron density of the arc-discharge plasma compared to the electron density in the sputtering process leads to the formation of a high-density plasma beam for sublimation of the IWO tablet. The picture in Fig. 1a shows the curved plasma beam in standby mode prior to the IWO tablet sublimation mode. The most sublimated gases are positively ionized by passing the high-density plasma beam region. Then, the positively ionized gases are strongly repelled from IWO tablet and accelerated to the self-biased PET substrate. The high energy of the accelerated ions results in enhancement of adhesion and densification of films as well as crystallization even at room temperature. Figure 1b shows pictures of the transparent and flexible ion-plated IWO/PET sample and flexible OLED and QDLED fabricated on the optimized flexible IWO/PET samples. Table 1 summarizes the Hall measurement results of 100 nm thick ion-plated IWO films on and 100 nm thick sputtered ITO films on PET substrates. Even when prepared at room temperature, the ion-plated IWO film had a lower resistivity and sheet resistance than the sputtered ITO films. The low resistivity of the ion-plated IWO films could be attributed to the higher carrier mobility (59 cm^2^/V-s) of the IWO film compared to sputtered ITO films (22.6 cm^2^/V-s) in spite of the lower carrier concentration of the IWO films. Without *in situ* substrate heating or post annealing, the ion-plated IWO film showed high mobility due to densification and crystallization of accelerated ions on the flexible PET substrate. In our previous work, we reported that a sputtered IWO film at room temperature has a high resistivity of 1.16 × 10^−3^ Ohm-cm due to insufficient activation energy for the W dopant^34^. For the high resistivity of the sputtered IWO films prepared at room temperature, a post annealing process should be required for activation of the W dopant and crystallization of the films. However, the ion-plated IWO films showed a much lower resistivity than the sputtered IWO or ITO films, even at room temperature. During the ion plating process, the ionized W^6+^ or W^4+^ ions easily substitute the In^3+^ sites in the In_2_O_3_ matrix and produce excess electrons in the IWO films. In addition, the screening effect of the W dopant reduced the scattering of electrons and increased the carrier mobility, as experienced in high mobility transparent conducting oxides^22,34–44^. As suggested by Zhang *et al*., high mobility of the In_2_O_3_-based transparent conducting oxide film could be explained by high Lewis acid strength (LAS) of transition metal dopants such as Ti^4+^, Zr^4+^, Gd^3+^, Mo^6+^, and W^6+^^45,46^. The LAS for IWO and ITO film could be calculated from below equation^34,45,46^:

$$LAS=\frac{Z}{{R}^{2}}-7.7{X}_{z}+0.8$$

where Z is the charge number of the atomic core, R is the ion radius (W^6+^: 0.68 Å, Sn^4+^: 0.71 Å), and X_z_ is electronegativity of element. Therefore, the LAS of the W^6+^ dopant (3.15) is much higher than that of Sn^4+^ (0.228). The W^6+^ dopant with a higher LAS value than Sn^4+^ in In_2_O_3_-based TCOs can polarize electronic charge from the O^2−^: 2p^6^ valence band more strongly towards itself. This polarzied electronic charge resulted in screening of charge and increased carrier mobility in the ion plated IWO film. Therefore, the high mobility of ion plated IWO films could be explained by the high LAS of the W^6+^ dopant in the In_2_O_3_ matrix^34^.

**Table 1:** Hall measurement results of sputtered ITO (Reference) and ion plated IWO films on PET substrate prepared at room temperature.

	Sheet resistance (Ohm/square)	Resistivity (Ohm-cm)	Mobility (cm^2^/V-s)	Carrier concentration (cm^−3^)
Sputtered ITO	51.37	5.137 × 10^−4^	22.6	5.37 × 10^20^
Ion plated IWO	36.39	3.639 × 10^−4^	59	2.91 × 10^20^

**Figure 2:**
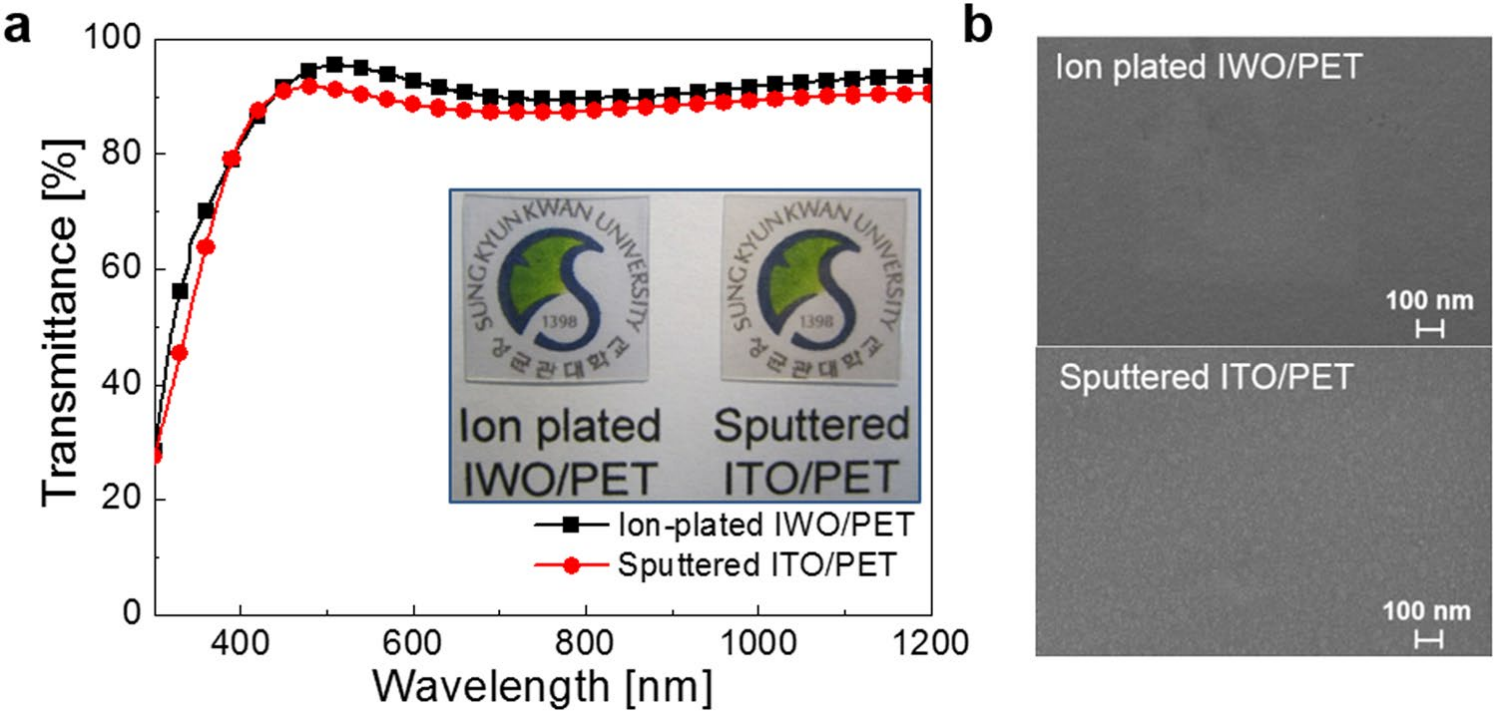
(**a**) Optical transmittance and (**b**) surface FESEM images of the ion-plated IWO and sputtered ITO films on PET substrates prepared at room temperature.

Figure 2a compares the optical transmittance of the ion-plated IWO films and sputtered ITO films grown on PET substrates, including the transmittance of the flexible substrate. The ion-plated IWO film possessed a high optical transmittance of 94.6% at a wavelength of 550 nm and an average transmittance of 91.4% between wavelengths of 400 to 800 nm, even when prepared at room temperature. However, the sputtered ITO film on a PET substrate showed a fairly reduced optical transmittance of 90.0% at a wavelength of 550 nm and an average transmittance of 88.6% between wavelengths of 400 and 800 nm. The picture in the inset compares the color and transparency of the ion-plated IWO/PET and sputtered ITO/PET samples. Both samples clearly show the Sungkyunkwan University logo due to the high optical transparency in the visible wavelength region. In addition, the ion-plated IWO film showed a slightly blueish color while the sputtered ITO films showed a slightly yellowish color. Due to the improved crystallinity of the ion-plated IWO films compared to sputtered ITO films, the ion-plated IWO films showed a much higher optical transmittance. From optical transmiitance of the IWO and ITO film, we can calclulated the bandgap (Eg) of the ion-plated IWO (3.85 eV) and sputtered ITO (3.6 eV) films. In spite of same In_2_O_3_ matrix elements, the ion plated IWO and sputtered ITO film showed different bandgap due to difference in crystallinity of room temperature processed IWO and ITO films. Figure 2b compares the surface FESEM images of the ion-plated IWO and sputtered ITO films on PET substrates. Unlike the sputtered ITO film with amorphous surface features, the ion-plated IWO film showed a very smooth and featureless surface image. Due to the different growth mode of the ion-plated IWO films and sputtered ITO films, the IWO films showed an atomically smooth surface morphology.

**Figure 3:**
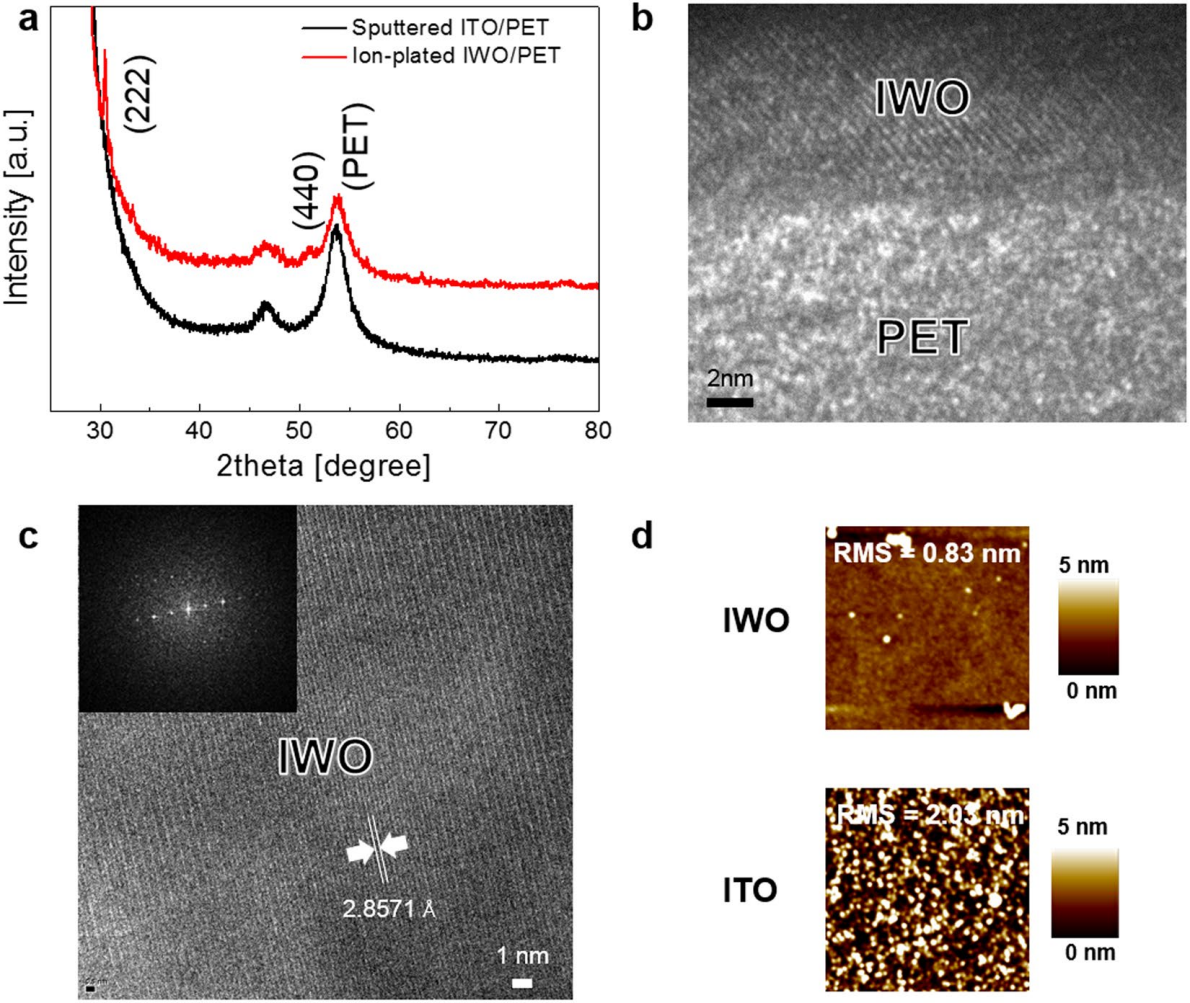
(**a**) XRD plots of the ion-plated IWO film and sputtered ITO films on PET substrates prepared at room temperature. (**b**) Enlarged image of the interface between the ion-plated IWO and PET substrate. (**c**) Cross-sectional TEM image obtained from the ion-plated IWO film where the inset shows the FFT pattern. (**d**) Surface AFM images of the ion-plated IWO films and sputtered ITO films.

Figure 3a shows the XRD plots of the ion-plated IWO film and sputtered ITO film prepared on PET substrates. The XRD plot of the sputtered ITO film exhibited only strong PET substrate peaks and no crystalline ITO peaks. Due to the room temperature sputtering process, the ITO/PET sample shows only broad PET substrate peaks due to the amorphous structure of sputtered ITO films^47^. However, the XRD plot of the ion-plated IWO films shows strong (222) and (440) peaks, indicating a well-developed crystalline structure in spite of the room temperature process. In particular, the stronger intensity of the (222) peak indicates that the ion-plated IWO film had a strong (222) preferred orientation. Figure 3b shows a cross-sectional TEM image obtained from the interface region between the ion-plated IWO and PET substrate. As expected from the XRD plot, the ion-plated IWO film on the PET substrate had a well-developed crystalline structure with a (222) preferred orientation even with the room temperature process. In particular, even in the initial growth region, the IWO films showed a crystalline structure. This is because the bombardment of accelerated ions into growing IWO films and the arrival of energetic ions on the PET substrate during the ion plating process lead to crystallization and densification of the IWO films. The crystalline IWO structure at the interface region indicates better adhesion of the ion-plated IWO film compared to typical sputtered ITO films. Figure 3c shows an enlarged TEM image obtained from the middle region of the ion-plated IWO film. Like the TEM image of the interface, the IWO film showed a (222) preferred crystalline structure. The d-spacing (d_222_ 222.8571 Å) of (222) plane is directly evaluated from high resolution TEM image. The strong spots in the FFT pattern in the inset also confirm the well-developed crystalline structure of the room temperature processed IWO film. Figure 3d compares the surface AFM images of the ion-plated IWO film and sputtered ITO film prepared at room temperature. Due to the room temperature process, both samples showed a low root mean square (RMS) roughness. However, the ion-plated IWO films showed a much lower RMS roughness of 0.83 nm than sputtered ITO films (2.03 nm) due to the absence of resputtering phenomena, which occurs in the sputtering process^48^. The smooth and featureless surface of TCE without protrusions, cracks, and holes is one of the important factors that affects the performance of flexible OLEDs and QDLEDs. In general, a hole transport layer (HTL) is directly coated on a TCE using a printing or evaporation process and the smoothness of the TCE critically affected the uniformity of the HTL and current spreading in the HTL layer.

**Figure 4:**
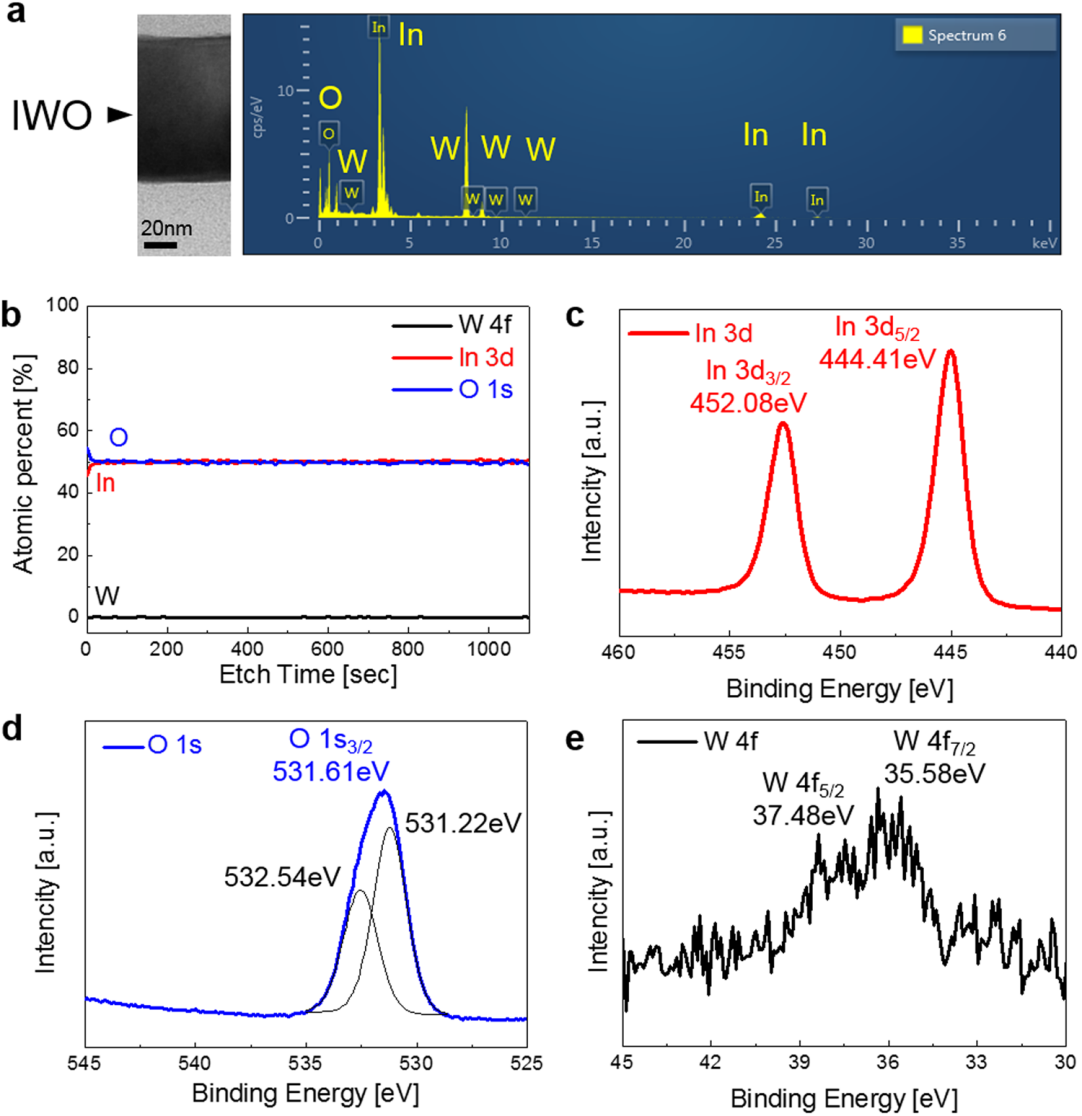
(**a**) EDS results of the ion-plated IWO film on a PET substrate. (**b**) X-ray photoelectron spectroscopy depth profile of the ion-plated IWO film. Core level spectra of (**c**) In 3d, (**d**) O 1 s, and (**e**) W 4 f obtained from the ion-plated IWO film.

To investigate the composition of the ion-plated IWO films, energy dispersive spectrometry (EDS) and X-ray photoelectron spectroscopy (XPS) analyses were employed. Figure 4a shows the EDS results obtained from the ion-plated IWO film on the PET substrate. The EDS result clearly shows In, O, and W peaks, indicating that the ion-plated IWO consisted of In, O, and W elements. The atomic composition of the ion-plated IWO film is 53.02% In, 46.03% O, and 0.95% W dopant. Compared to the In and O peak intensities, the peak intensity of the W dopant is very weak due to low doping content in the ceramic IWO tablet (1 at% W). Figure 4b exhibits the XPS depth profile of the ion-plated IWO film prepared at room temperature. The XPS depth profile shows constant In, O, and W atomic percentages with increasing etching time, indicating that the ion-plated IWO film had good compositional uniformity. In particular, W dopants were uniformly distributed throughout the In_2_O_3_ matrix. As expected from the EDS results, the IWO film showed a small atomic W dopant percentage. The binding energy of the ion-plated IWO film was analyzed by examining the core level spectra of In 3d, O 1 s, and W 4 f, as shown in Fig. 4(c–e). In Fig. 4c, the In 3d spectrum consists of a doublet with binding energies of 444.41 eV for In 3d_5/2_ and 452.08 eV for In 3d_3/2_, corresponding to the binding energies of the In^3+^ ion in In_2_O_3_^37^. In Fig. 4d, the peak with a binding energy of 531.61 eV for O 1 s_3/2_ is detected in the spectrum of O 1 s. In Fig. 4e, compared to the In 3d and O 1s peaks, the W 4 f peak has a weaker peak intensity due to the low doping concentration of W (1 at%) in In_2_O_3_. The O 1 *s* peak in Fig. 4d exhibits multiple components at binding energies of 531.22 and 532.54 eV. These deconvoluted two levels of O1*s* are closely related to the two types of O_2_ ions in the IWO films. The lower binding energy peak (O_II_) is from O_2_ ions that neighbor In atoms with their full complement of electrons, while the higher binding energy peak (O_I_) corresponds to oxygen vacancies in the IWO film like typical In_2_O_3_ based TCO electrodes^27^.

**Figure 5:**
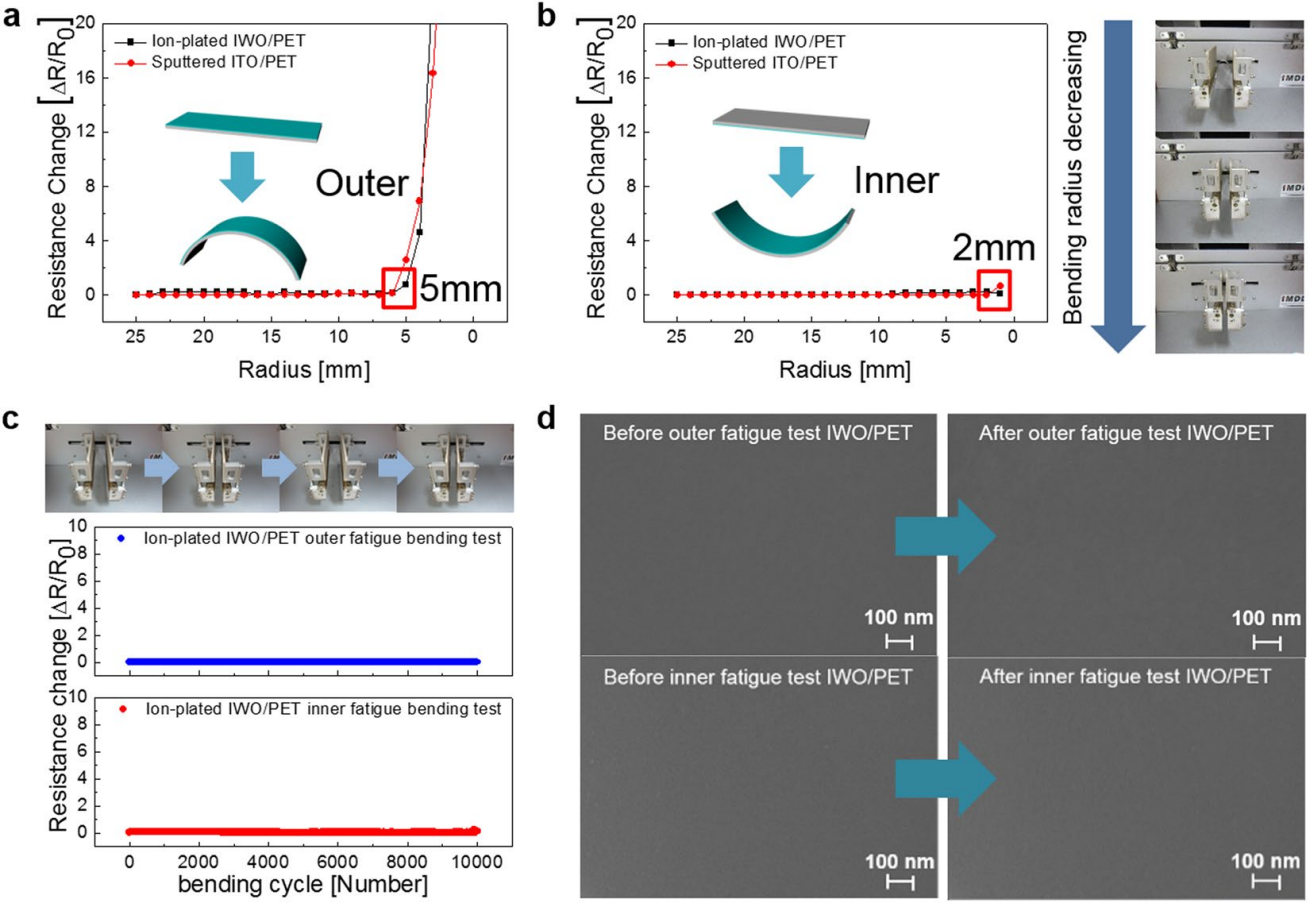
(**a**) Outer and (**b**) inner bending testing of ion-plated IWO/PET and sputtered ITO/PET samples with decreasing bending radius. The pictures on the right side show the outer and inner bending steps. In addition, the inset shows the tensile and compressive strains applied on the IWO films. (**c**) Dynamic outer and inner bending fatigue test results of ion-plated IWO/PET. (**d**) Surface FESEM images before and after 10,000 cycles of outer and inner bending of the ion-plated IWO/PET.

Figure 5 shows a comparison of the mechanical flexibilities of the ion-plated IWO films and sputtered ITO films on PET substrates. In the operation of flexible OLEDs and QDLEDs, the constant resistance change of TCE against outer bending and inner bending of the devices is very important. Therefore, we measured the resistance change during outer and inner bending of the samples. Figure 5a shows the outer bending test results of the ion-plated IWO and sputtered ITO films with decreasing outer bending radius. The pictures on the right side show the outer bending steps using our lab-scale bending machine. By pressing a clamped sample on both sides, we can decrease the bending radius of the sample. The change of the resistance of the ion-plated IWO films can be expressed as (Δ*R* nd*R* − *R*_0_)/*R*_0_, where *R*_0_ is the initial measured resistance and *R* is the *in situ* measured resistance under substrate bending. Up to an outer bending radius of 6 mm, both the ion-plated IWO/PET and sputtered ITO/PET samples showed a constant resistance change, indicating no crack formation. However, at an outer bending radius of 5 mm, both samples showed an abrupt increase of resistance change due to crack formation and separation of the IWO and ITO films on the PET substrate. Therefore, we determined that the critical outer bending radius of the ion-plated IWO film is 5 mm, which is similar to that of ITO films. The similar critical outer bending radii of the crystalline IWO and amorphous ITO films could be explained to be a result of similar strains. The following equation shows the peak strain for flexed IWO and ITO films with decreasing bending radius^49^.

$${\rm{Strain}}=\frac{{d}_{IWO}+{d}_{PET}}{2R}\times 100$$

Here, *d*_*IWO*_ and *d*_*PET*_ are the thicknesses of the IWO film and PET substrate, respectively. Bending of the 100 nm thick IWO film on a 125 µm thick PET substrate to a critical outer bending radius of 5 mm resulted in a peak strain of 1.25%. With such a high peak strain, regardless of structure, typical oxide films are easily cracked and separated. Therefore, at an outer bending radius of 5 mm, both samples showed an abrupt increase of the resistance change, as confirmed by the FESEM images. Compared to outer bending, the inner bending test results shown in Fig. 5b reveal a lower critical bending radius of 2 mm. As shown in the inset, during the inner bending test, the IWO films experienced compressive strain, unlike in the outer bending test. Therefore, the cracks or laminated films were physically contacted or overlapped. Due to physical contact of the cracked films, there is no change of the resistance until a bending radius of 2 mm. Therefore, we determined that the ion-plated IWO film is stable at a bending radius of 10 mm, regardless of the bending mode. At a fixed bending radius of 10 mm, the IWO films experienced repeated outer and inner bending cycles, as shown in Fig. 5c. The picture in the inset shows the dynamic fatigue test steps. Both dynamic outer and inner bending fatigue tests showed no change of the resistance (Δ*R*) after 10,000 bending cycles at the fixed bending radius of 10 mm. Figure 5d shows surface field emission scanning electron microscope (FESEM) images of the ion-plated IWO/PET film after 10,000 cycles of the dynamic outer/inner bending fatigue tests. Even after 10,000 cycles, the IWO film on the PET substrate showed a very clean surface without cracks or surface defects, similar to the as-deposited IWO films.

**Figure 6:**
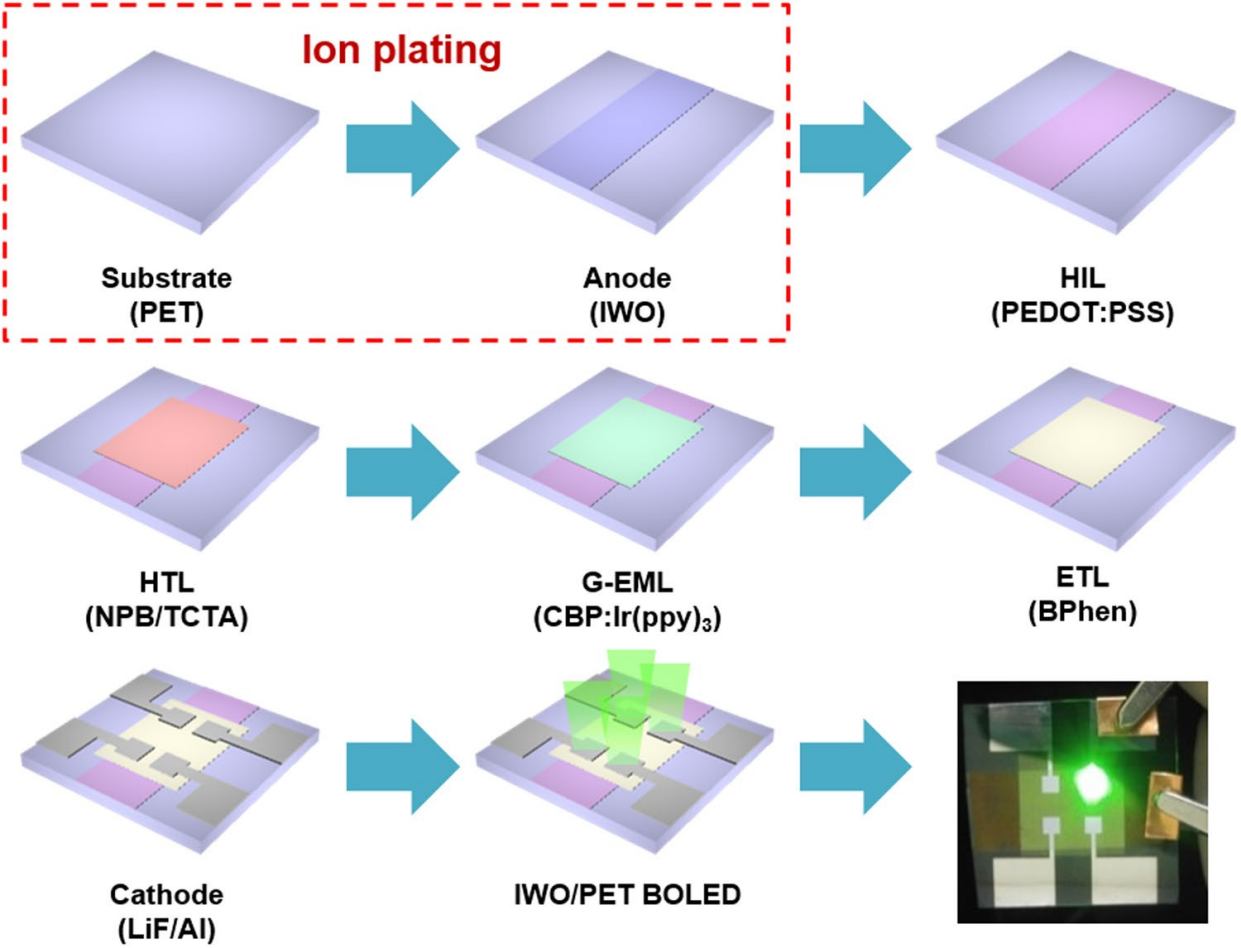
Schematic fabrication process of the flexible OLED on a patterned ion-plated IWO anode and picture of the green light emitting flexible OLED with an IWO anode.

To show the potential of the room temperature processed IWO anode, we fabricated a flexible OLED with the patterned IWO electrode as an anode. Figure 6 is a schematic diagram showing the fabrication procedure of the flexible OLED on the ion-plated IWO electrode. The HIL layer was spin-coated on the patterned IWO anode. Then, the organic HTL, G-EML, ETL, and LiF/Al cathode were evaporated in a vacuum evaporator.

**Figure 7:**
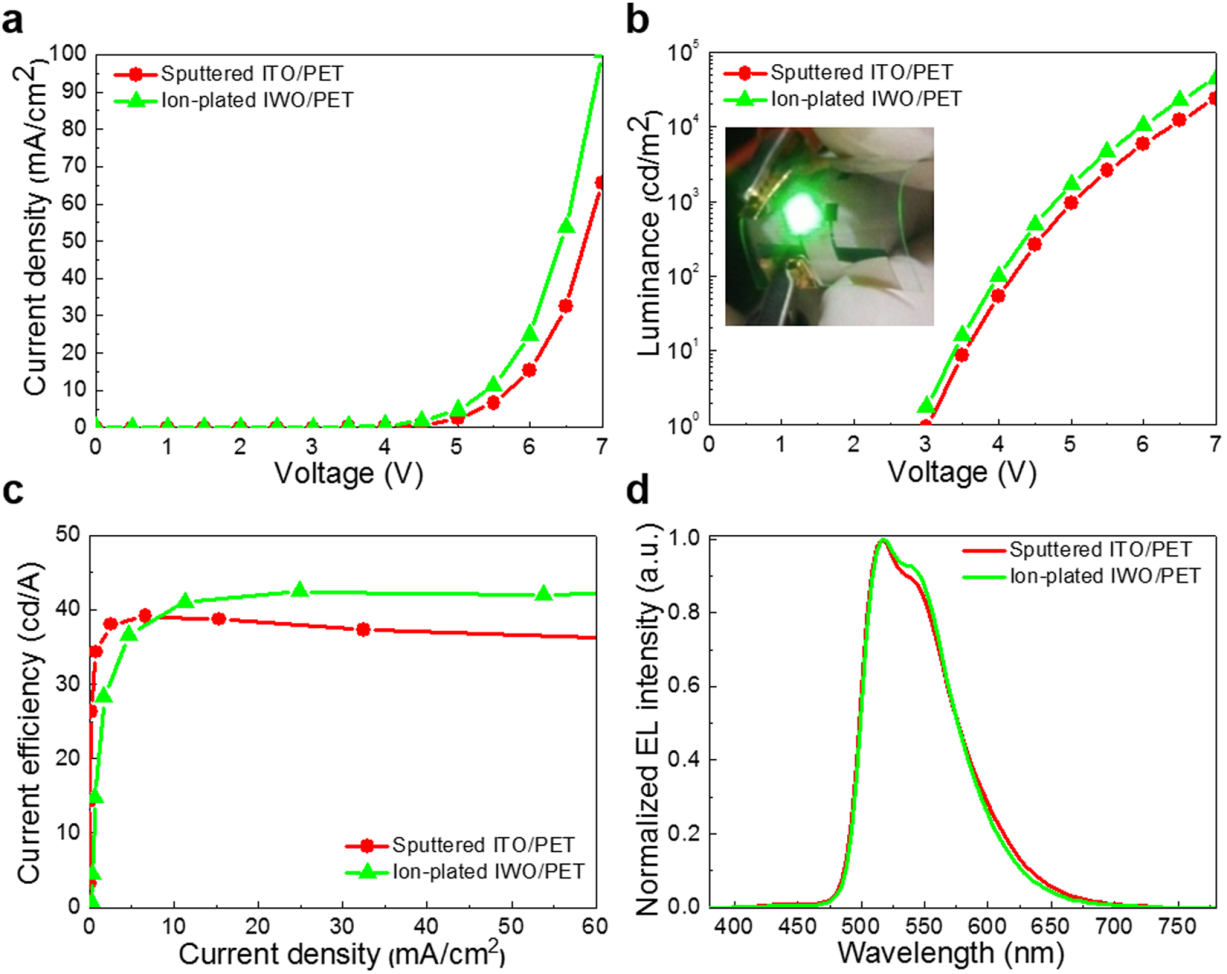
(**a**) Current density-voltage curve, (**b**) luminance-voltage curve, (**c**) current efficiency–current density curve, and (**d**) EL spectra of flexible OLEDs fabricated on ion-plated IWO and sputtered ITO anodes. Both samples were fabricated in the same chamber used to deposit the organic layers and metal cathode. The inset picture in (**b**) shows green light emission from the flexible OLED with the ion-plated IWO anode.

Figure 7a and b show the current density-voltage (J-V) and luminance-voltage (L-V) characteristics of the flexible OLEDs fabricated on the ion-plated IWO and sputtered ITO anodes. The flexible OLEDs with IWO and ITO anodes had similar turn-on voltages of 3.0 and 3.0 V, respectively. Due to the low sheet resistance and high optical transmittance, the flexible OLED with the ion-plated IWO anode showed a higher current density and luminance after turn-on than the flexible OLED with the sputtered ITO anode. The L-V curves of the flexible OLEDs with IWO and ITO anodes also showed similar steep increases after onset. The inset picture shows an image of the light emission pixels from the flexible OLED with an ion-plated IWO anode. In particular, the flexible OLED fabricated on the ion-plated IWO anode exhibited a higher current efficiency at a higher current density and better roll-off behavior, as shown in Fig. 7c, due to the low sheet resistance and high optical transmittance of the IWO anode. Due to the same thicknesses of the IWO and ITO anodes, the EL spectra of the flexible OLEDs are similar, as shown in Fig. 7d. However, the OLED with the ion-plated IWO anode showed a slightly higher intensity in the wavelength region of 550 nm due to the higher optical transmittance of the IWO anode. Consequently, the better device performances of the flexible PSCs and OLEDs on the ion-plated IWO electrode compared to the sputtered ITO electrode demonstrates the potential of the ion-plated IWO electrode as a promising FTCE.

**Figure 8:**
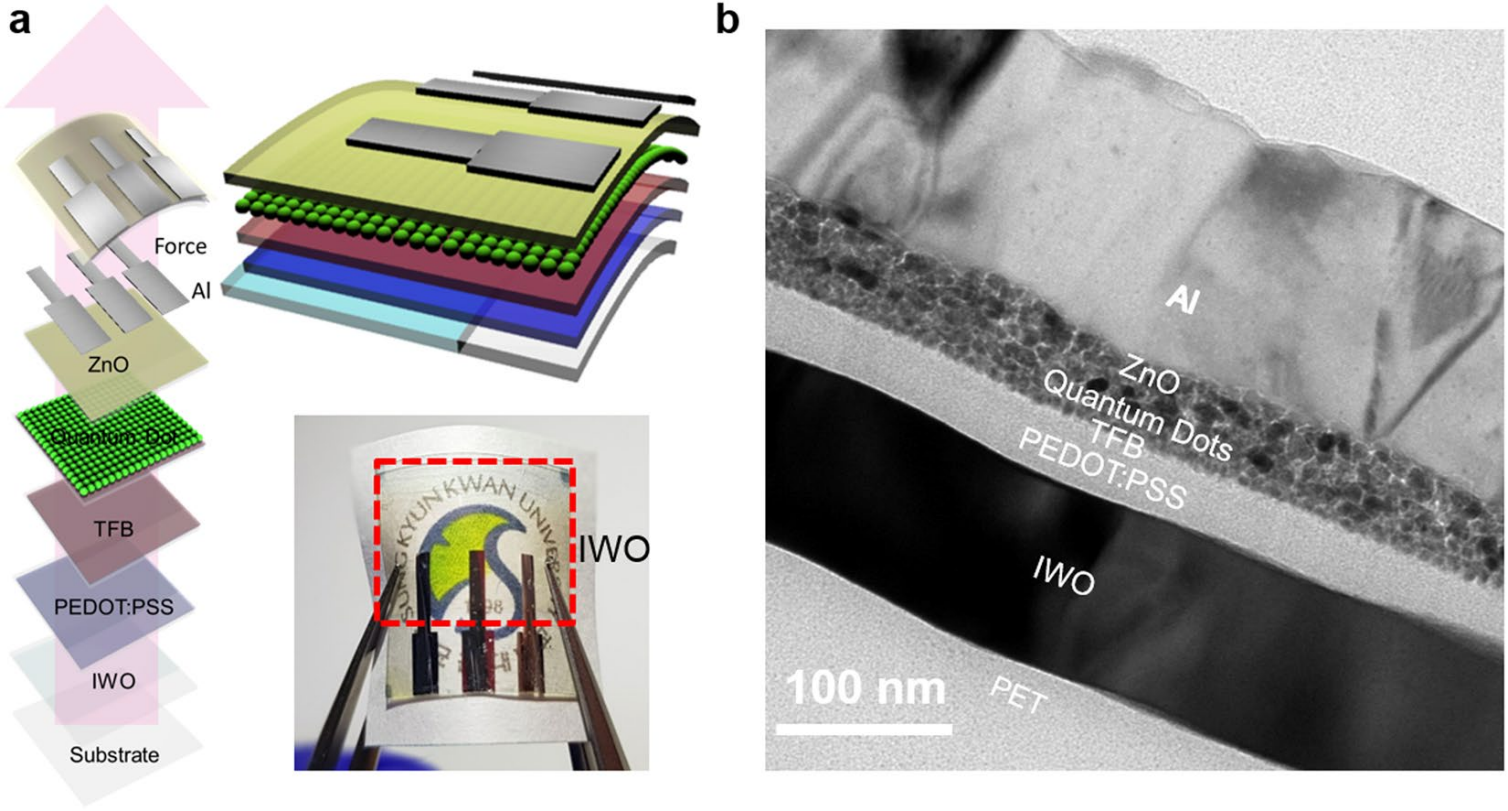
(**a**) Fabrication process of the QDLED on the ion-plated IWO/PET sample. The dashed region in the picture indicates the patterned IWO anode. (**b**) Cross-sectional TEM image of the QDLED on the ion-plated IWO anode.

To apply the ion-plated IWO film as a flexible and transparent anode in the fabrication of QDLEDs, we fabricated typical QDLEDs on the ion-plated IWO and sputtered ITO. Figure 8a shows a schematic fabrication process of the flexible QDLED structure on the IWO anode. The QDLEDs were fabricated on the IWO and reference ITO anodes following our standard QDLED fabrication steps^50^. The dashed line in Fig. 8a represents the patterned ion-plated IWO anode. Figure 8b shows a cross-sectional TEM image of the QDLED with the ion-plated IWO anode. As expected from the AFM image, all PEDOT:PSS/TFB/CdSe QDs/ZnO/Al layers were uniformly coated on the ion-plated IWO anode without intermixing or protrusions due to the atomic scale smoothness of the IWO anode.

**Figure 9:**
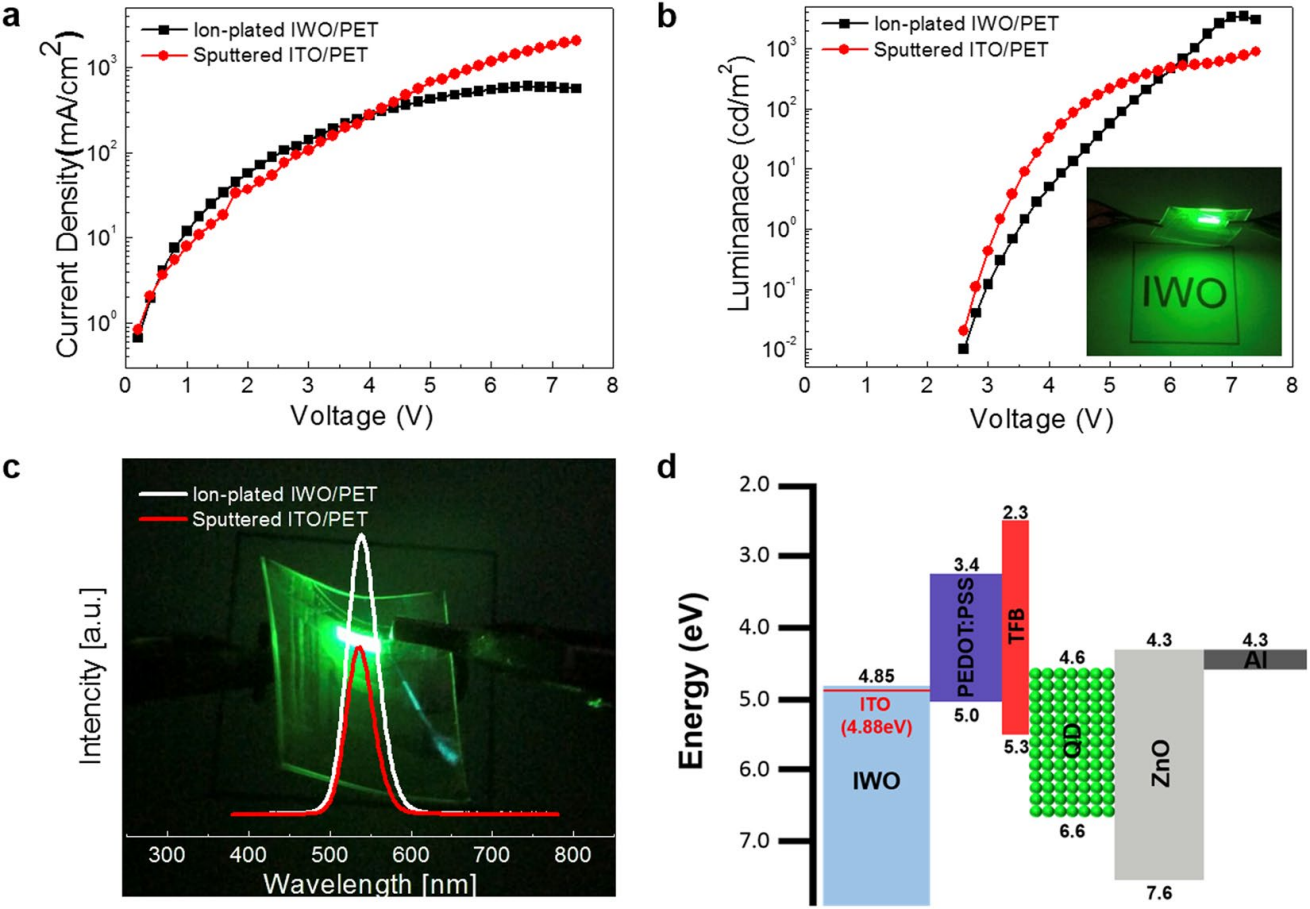
(**a**) Current density-voltage (J-V) curve, (**b**) luminance-voltage (L-V) curve, (**c**) intensity-wavelength curve, and (**d**) energy level diagram for flexible QDLEDs with ion-plated IWO and sputtered ITO electrodes at room temperature. The inset pictures in (**b**) and (**c**) show green light emission from the flexible QDLED with the ion-plated IWO anode.

Figure 9a shows the current density-voltage (J-V) characteristics of the flexible QDLEDs fabricated on the ion-plated IWO and sputtered ITO anodes. Due to the lower sheet resistance and higher optical transmittance of the IWO anode compared to those of the sputtered ITO anode, the flexible QDLED made with the IWO anode showed a slightly higher current density after turn-on of the flexible QDLEDs. Although the flexible QDLEDs with IWO and ITO anodes had similar turn-on voltages of 3.6 and 3.2 V, respectively, due to similar work functions and good Ohmic contact properties between the oxide anodes and the PEDOT:PSS HTL layer, the flexible QDLED with the sputtered ITO anode quickly died below a voltage of 5 V. Due to ineffective hole injection through the amorphous ITO with a higher sheet resistance, the flexible QDLED with the ITO anode cannot stand the input voltage of 5 V. In particular, unstable interface between the amorphous ITO and PEDOT:PSS HTL led to ineffective hole injection. The luminescence-voltage curves of the flexible QDLEDs with IWO and ITO anodes also demonstrated similar steep increases after onset as shown in Fig. 9b. However, the flexible QDLED with the IWO anode exhibited a higher luminance in sphite of lower current density compared to the ITO-based QDLED at a high operation voltage region above 6 V. Due to effective hole injection through the ion plated IWO electrode, the flexible QDLED showed higher luminance than flexible ITO-based QDLED. Figure 9c compares the electroluminance spectra of the flexible QDLEDs with the ion-plated IWO and sputtered ITO anodes. To understand the hole carrier injection, the work functions of the ion-plated IWO and sputtered ITO anode were measured using a Kelvin probe force microscopy (KPFM) system. The work functions of the ion-plated IWO and sputtered amorphous ITO are 4.85 ± 0.01 eV and 4.88 ± 0.01 eV, respectively. Based on the work functions of the ion-plated IWO and sputtered ITO anodes, band diagrams of the flexible QDLEDs are illustrated in Fig. 9d. Due to the similar band diagrams, the poor performance of QDLEDs with the ITO anode could be explained to be due to the unstable interface and fast degradation of the amorphous ITO anode during operation of the QDLED.

**Figure 10:**
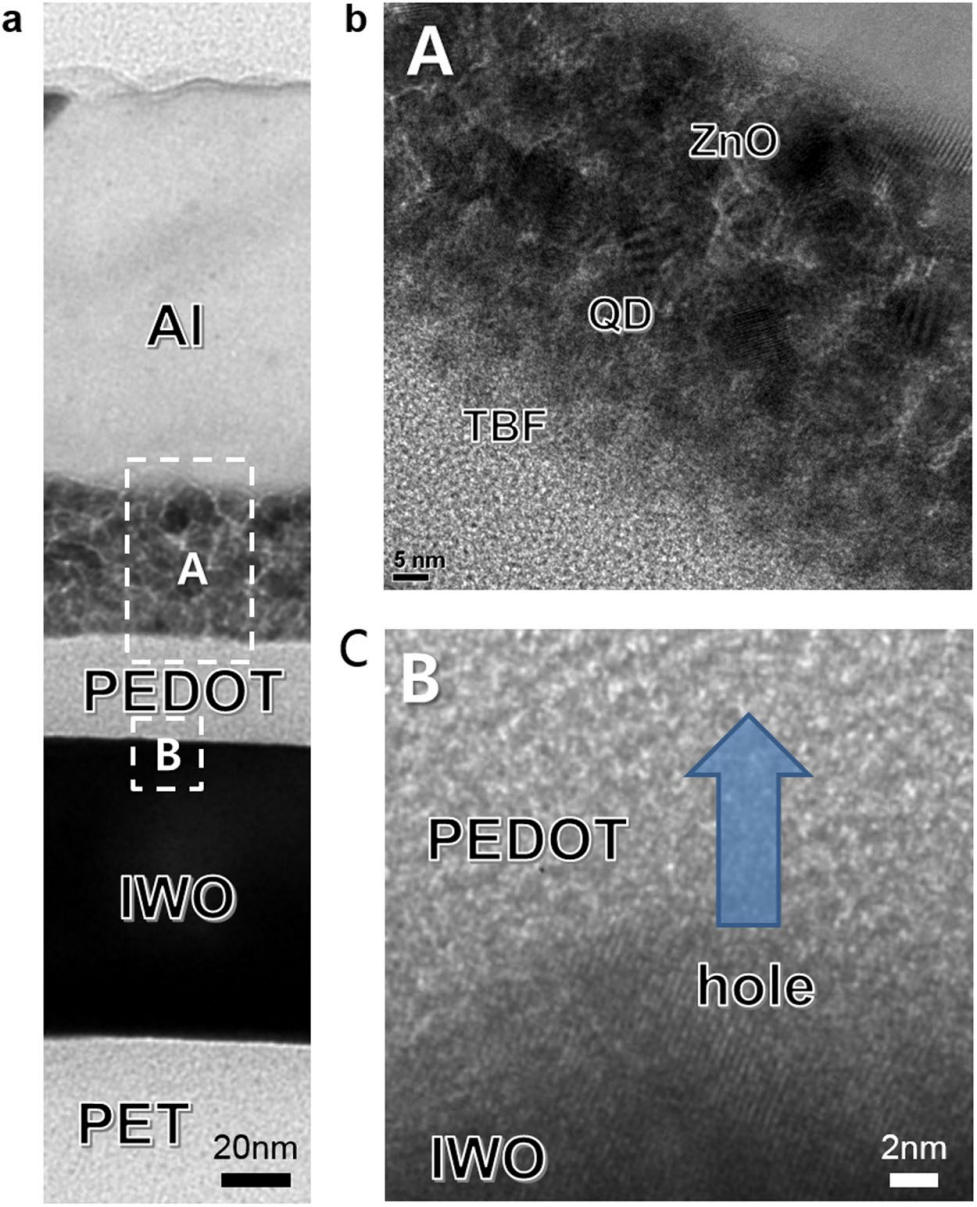
(**a**) Cross-sectional TEM image of a flexible QDLED with an ion-plated IWO anode. (**b**) Enlarged HRTEM obtained from the lettered region from A in the cross-sectional image. (**c**) Enlarged HRTEM obtained from the lettered region from B in the cross-sectional image.

To investigate the interface between the ion-plated IWO and PEDOT:PSS, we utilized TEM, as shown in Fig. 10. The cross-sectional TEM image in Fig. 10a shows a well-defined IWO anode, PEDOT:PSS layer, QD active layer, and Al cathode. The bright field image of the IWO anode shows a very flat and atomic scale smooth surface even though it has well-developed crystallinity. In particular, the interface between the IWO anode and PEDOT:PSS was very clean and no interfacial reactions occurred. Unlike the rough surface morphology generally found in sputtered ITO films, the ion-plated IWO film showed a very good smooth surface morphology because there are no subgrains formed by the resputtering effect^46^. The gray contrast of the PEDOT:PSS layer indicates the amorphous structure of the PEDOT:PSS layer. Even though the IWO anode has a similar work function (4.85 eV) as the sputtered ITO anode (4.88 eV), the QDLEDs with IWO and ITO anodes showed similar turn-on voltages, probably due to the presence of the amorphous PEDOT:PSS buffer layer between the anode. The cross-sectional TEM image of the flexible QDLED with the IWO anode also shows a stable interface between the IWO anode and the PEDOT:PSS layer. The amorphous PEDOT:PSS layer was in good contact with the IWO anode. Figure 10b and c are enlarged TEM images of the “A” and “B” regions, respectively, in the cross-sectional TEM image. The enlarged TEM image from the QD active layer (A region) shows that TBF and the QD and ZnO nanoparticle layer were well contacted without protrusions or voids. Note that good contact was established between the PEDOT and QD active layer in the ‘A’ region. The IWO anode below the PEDOT HTL layer region in ‘B’ showed a well-developed bixbyite structure with a (222) preferred orientation. Due to the low sheet resistance of the IWO anode, the hole carriers easily flow into the PEDOT:PSS HTL layer, as indicated by the arrow. In typical QDLEDs, efficient injection of the hole carrier from the anode to the QD active layer is difficult due to the mismatched work function, as shown in Fig. 9d. Therefore, an additional acidic PEDOT:PSS buffer layer with a high work function is necessary for hole carrier injection from the anode layer. Consequently, the well-matched and stable interface between the crystalline IWO and PEDOT:PSS HTL layer led to better performance of the flexible QDLEDs compared to ITO-based QDLEDs.

## Discussion

We investigated the characteristics of IWO films processed at room temperature on a flexible PET substrate using an in-line arc plasma ion plating system and compared the results to sputtered ITO films prepared at room temperature. Due to high energy flux of sublimated ions generated from the plasma region, the ion-plated IWO films showed a well-developed crystalline structure, had a low sheet resistance of 36.39 Ohm/square, and an optical transmittance of 94.6% even though they were prepared at room temperature. The low sheet resistance of the room temperature processed IWO film is attributed to the high mobility (59 cm^2^/V-s) in a crystalline IWO film and screening effect of the W dopant. In particular, the ion-plated IWO film showed a high optical transmittance in the visible wavelength region due to good crystallinity. In addition, good adhesion of the ion-plated IWO film on the PET substrate resulted in small critical outer and inner bending radii of 6 and 3 mm against substrate bending, respectively, which is an important factor that affects the performance of flexible OLED and QDLEDs. Due to the high optical transmittance, low sheet resistance, and outstanding flexibility of the ion-plated IWO films, the flexible OLEDs and flexible QDLEDs with the IWO electrodes showed better performances than ITO-based flexible OLEDs and QDLEDs. This indicates that in-line arc plasma ion plating is a promising coating technique to realize room temperature processed high-quality IWO for flexible OLEDs and QDLEDs.

## Methods

### In-line ion plating process for room temperature processed IWO electrodes

Flexible and transparent IWO films were deposited at room temperature on PET substrates using an ion plating system without breaking vacuum. At a working pressure of 3 mTorr in the vacuum chamber, the 100 nm thick IWO films were deposited onto the PET substrate using an IWO tablet (99 at% In_2_O_3_-1 at% W). The operating conditions included a power of 3,000 W, an Ar/O_2_ flow rate of 300/90 sccm, a process speed of 0.492 m/min, and a dynamic deposition rate of 541.2 Å/min. The distance between the tablets and the PET substrate was maintained at 600 mm.

### Characterization of the ion-plated IWO electrodes

The optical properties of the ion-plated electrodes were examined using a UV/visible spectrometer (Unicam, UV 540). The electrical properties of the ion-plated electrodes were measured at room temperature by means of Hall measurement (Accent optical technology, HL5500PC) with van der Pauw geometry. The structural properties of the samples were examined using X-ray diffraction (XRD, Bruker, D8 Advance) and X-ray photoelectron spectroscopy (XPS, Thermo Electron, K-Alpha). The surface morphology and work function of the samples was examined using Kelvin probe force microscopy (KPFM, Park system, XE-100). In addition, the microstructural and surface properties of the ion-plated electrodes were analyzed using field emission scanning electron microscope (FESEM, Carl Zeiss, MERLIN) and high resolution transmission electron microscopy (HRTEM, JEOL, JEM-2100F). The mechanical integrity of the ion-plated electrodes was evaluated using a lab-made inner/outer bending machine. In addition, dynamic fatigue bending tests were performed using a lab-designed cyclic bending test machine operated at a fixed bending radius of 10 mm.

### Fabrication and evaluation of flexible organic light emitting diodes

The IWO anode was patterned by a shadow mask attached on a PET substrate during arc plasma ion plating. Then, a 30–40 nm thick hole injection layer (HIL) (PEDOT:PSS, Heraeus, Clevios PVP AI 4083) was spin-coated and 40 nm thick hole transport layers (HTLs) were vacuum-deposited (N,N′-Bis(naphthalen-1-yl)-N,N′-bis(phenyl)benzidine (TAEWON SCIENTIFIC CO., LTD, NPB) (30 nm) and 4,4′,4″-Tris(carbazol-9-yl)triphenylamine (DAEYEON CHEMICALS CO., LTD, TCTA) (10 nm)), which provide a lower driving voltage, reduced leakage current, and high triplet energy level for a stable OLED. Next, a 30 nm thick 7 wt% Tris[2-phenylpyridine]iridium(III) (DAEYEON CHEMICALS CO., LTD, Ir(ppy)_3_)-doped 4,4′-Bis(N-carbazolyl)-1,1′-biphenyl (OSM, CBP) layer was evaporated as a green phosphorescent light emitting layer (G-EML). Then, 25 nm of Bathophenanthroline (OSM, BPhen) was used as the electron transport layer (ETL). Finally, LiF (0.5 nm) and a 100 nm thick aluminum cathodes were sequentially deposited. The electroluminescence (EL) characteristics of the device were measured by a conventional current-voltage-luminance (I-V-L) measurement system (Photo Research Inc., PR-655).

### Fabrication and evaluation of flexible quantum dot light emitting diodes

The device consisted of a PET substrate, sputtered ITO (100 nm) or ion-plated IWO (100 nm) as anodes, PEDOT:PSS (Heraeus, Clevios PVP AI 4083) (30 nm) as a hole injection layer (HIL) on the UV-ozone-treated anode, Poly[(9,9-dioctylfluorenyl-2,7-diyl)-co-(4,4A-(N-(4-sec-butylphenyl)) diphenylamine)] (Lumtec, TFB) (3 nm) as a hole transporting layer (HTL), Colloidal CdSe QD (Nanosquare, NSQDs-HOS) (13 nm) layer as an emitting layer, a ZnO (Avantama, N10) (35 nm) layer as an electron transporting layer (ETL), and a Al metal cathode (130 nm). The 2-propanol solvents are used for coating of ZnO on QD layers. All layers except the electrodes of the QDLEDs were coated using a spin coating method. On the other hand, the Al used as the cathode of QDLEDs was deposited through a patterned shadow mask using a thermal evaporator. The electroluminescence (EL) characteristics of the device were measured by a conventional current-voltage-luminance (I-V-L) measurement system (McScience, M6100).

### Manuscript comments

One of the co-author (Jae-Gyeong Kim) drew drawings in Figure 1a, inset of Figure 5a and b, Figuer 6, and Figuer 8a using a **RHINO** drawing program. The url link of the **RHINO** drawing program is http://www.rhino3d.com. In addition, we checked the terms of use of the software **RHINO**. In case of Figure 9d, it was drewn by Microsoft PowerPoint 2013.
